# Prognostic Impact of Tumor Budding on Moroccan Colon Cancer Patients

**DOI:** 10.1155/2022/9334570

**Published:** 2022-01-21

**Authors:** Fatima El Agy, Sanae el Bardai, Laila Bouguenouch, Nada Lahmidani, Mohammed El Abkari, El Bachir Benjelloun, Abdelmalek Ousadden, Khalid Mazaz, ImaneToughrai, Sidi Adil Ibrahimi, Zineb Benbrahim, Laila Chbani

**Affiliations:** ^1^Laboratory of Biomedical and Translational Research, Faculty of Medicine and Pharmacy, Sidi Mohamed Ben Abdellah University, Fez, Morocco; ^2^Laboratory of Anatomic and Molecular Pathology, University Hospital Hassan II, Fez, Morocco; ^3^Laboratory of Medical Genetics and Oncogenetics, University Hospital Hassan II, Sidi Mohamed Ben Abdellah University, Fez, Morocco; ^4^Department of Gastroenterology, University Hospital Hassan II, Sidi Mohamed Ben Abdellah University, Fez, Morocco; ^5^Department of General Surgery, University Hospital Hassan II, Sidi Mohamed Ben Abdellah University, Fez, Morocco; ^6^Department of Oncology, University Hospital Hassan II, Sidi Mohamed Ben Abdellah University, Fez, Morocco; ^7^Laboratory of Anatomic and Molecular Pathology, University Hospital Hassan II, Sidi Mohamed Ben Abdellah University, Fez, Morocco

## Abstract

**Background:**

Tumor budding is now emerging as one of the robust and promising histological factors that play an important role in colon cancer. In this study, we aimed to investigate the association between tumor budding and tumor clinicopathological factors, tumor molecular signature, and patient survival for the first time in a Moroccan population.

**Methods:**

We collected data of 100 patients operated from colon adenocarcinoma. Tumor budding was assessed on HES slides, according to the International Tumor Budding Consensus Conference 2016 recommendations. The expression of MMR proteins was performed by immunohistochemistry. KRAS and NRAS mutations testing was performed by Sanger sequencing and pyrosequencing.

**Results:**

High tumor budding grade (BUD 3) was found to be significantly associated with adverse clinicopathological features including older age (*P*=0.03), presence of perineural invasion (*P*=0.02), presence of vascular invasion (*P*=0.05), distant metastases (*P* < 0.001), advanced TNM stage (*P*=0.001), the occurrence of relapse (*P*=0.04), and the high number of deceased cases (*P*=0.02). Interestingly, we found that tumors with high-grade tumor budding were more likely to be microsatellite stable (MSS) (*P*=0.005) and harbor more KRAS mutations (*P*=0.02). Tumors with high-grade tumor budding were strongly associated with KRAS G12D mutation (*P*=0.007). In all stages, high tumor budding was correlated with poorer overall survival (*P*=0.04) and decreased relapse-free survival with a difference close to significance ((*P*=0.09). We concluded that high tumor budding was strongly associated with unfavorable clinicopathological features and special molecular biomarkers and effectively affects the overall survival of CC patients.

**Conclusions:**

Based on these findings and the ITBCC group recommendations, tumor budding should be taken into account along with other clinicopathologic factors in the risk assessment of colorectal cancer.

## 1. Introduction

Colon cancer is the third most commonly diagnosed cancer and the second leading cause of cancer-related death in the Moroccan population [1]. A higher incidence of this disease occurs consistently among males than females in the general population with a median age at diagnosis of 55.56 years [2].

The tumor node metastasis (pTNM) stage is the primary factor used for prognostication purposes and to guide patient management [3]. Indeed, the standard of care for colon cancer is surgical resection (stages I and II), and surgery is followed by adjuvant chemotherapy (fluorouracil and folinic acid) for stage II with high-risk factors tumors. Neoadjuvant chemotherapy and targeted therapies (anti-EGFR) are used for colon cancer with distant metastasis. Although and because of the survival heterogeneity seen in colon cancer patients within the same pathological stage, the introduction of other molecular, immunological, and histological markers to identify risk stratification and disease outcome is now mandatory for better management of colon cancer patients.

Tumor budding is now emerging as one of the robust and promising histological factors that play an important role in colon cancer. It is a histological manifestation of initiating invasion and metastasis cascade in the invasive front of the tumor. According to the International Tumor Budding Consensus Conference 2016, ITBCC, it is defined by the presence of individual cells and small clusters (<5) of tumor cells at the invasive front of carcinomas [4]. Several studies have demonstrated that tumor budding might be associated with a high risk of relapse and poorer outcomes [5]. Its relationship with unfavorable clinicopathological features like nodal and distant metastases was also demonstrated [4, 5]. Indeed, higher grade of tumor budding was reported to be significantly correlated with microsatellite stable tumors (MSS tumors) [6, 7]. Furthermore, Sammarco et al. have recently demonstrated that microsatellite “stable” BRAF-mutated tumors show more aggressive morphological behavior like tumor budding [8].

Recently, a study conducted by Anne et al. on 1320 colon cancer patients has shown that high tumor budding was associated with the presence of KRAS mutations and metastatic tumors harboring a BRAF gene mutation [9].

Several studies have revealed the clinical implication of tumor budding in colon cancer management. First, in colon cancer stage I, tumor budding is associated with lymph node metastasis. For this reason, patients with high tumor budding may benefit from oncologic resection [4]. Second, in stage II colon cancer, the presence of tumor budding is associated with poorer survival. Therefore, adjuvant therapy should be discussed for stage II colon cancer patients with high-grade tumor budding [10]. Third, the assessment of tumor budding in preoperative biopsies could be useful for selecting patients who may qualify for neoadjuvant therapy. However, in advanced colon cancer, the role of tumor budding in clinical practice remains unclear and requires more investigation [11].

The lack of a standard quantification method for tumor budding has limited its reporting in the clinical routine practice in CC as well as other histological factors. However, after the International Tumor Budding Consensus Conference (ITBCC), held in Bern in April 2016, a scoring system for assessing tumor budding has been reached according to conference recommendations [4]. The ITBCC group recommended that tumor budding should be included in guidelines/protocols and staging systems for the pathology reporting of colorectal cancer [4].

This study aimed to assess tumor budding according to the ITBCC recommendations and to investigate the association between tumor budding and tumor clinicopathological factors, tumor molecular signature, and patient's survival for the first time in a Moroccan population.

## 2. Materials and Methods

### 2.1. Patients

**Figure 1:**
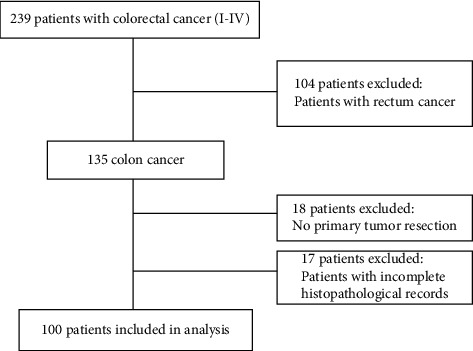
Flow diagram for the study.

We enrolled in the present study a hundred patients with primary colon cancer resected between 2015 and 2020, at Hassan II University Hospital, Fez, Morocco. The medical charts were prospectively and retrospectively reviewed and patients were included according to the following inclusion criterion: patients with histologically confirmed primary adenocarcinoma, all cases with I-IV stage colon cancer, and patients with prognostic data. Patients were excluded from this study due to the following exclusion criteria: All patients with incomplete clinical records, patients without histological confirmation of colon adenocarcinoma, and patients with rectal cancer (Figure 1). Demographic and clinicopathological data (e.g., age, gender, tumor grade, tumor localization histological subtype, tumor grade, disease stage, and number of examined regional lymph nodes) and follow-up data were collected from the patient's medical records and pathology reports.

### 2.2. Pathology Analysis

After first-line therapy, fresh specimens were transported to the department of pathology. The tissue was fixed in formalin (10%) and embedded in paraffin (FFPE). Histological slides based on hematoxylin and eosin staining were prepared and examined by a pathologist to define the histopathological characteristics of the tumor.

### 2.3. Assessment of Tumor Budding

Tumor budding was assessed on hematoxylin-eosin and Safran (HES) stained slides. For each CC case, one representative HES slide was used for scoring by the pathologist according to the ITBCC recommendations. Tumor buds were evaluated in a single hotspot measuring 0.785 mm^2^ at the invasive front using microscopy at 20× objectives.

We then used a three-tier system which is recommended by the ITBCC group to provide tumor budding count and tumor budding category. The system of scoring is categorized as follows:

- 0–4 buds: low budding (Bd 1)
- 5–9 buds: intermediate budding (Bd 2)
- 10 or more buds: high budding (Bd 3)

We grouped the patients to be low-intermediate (grade 1 + grade 2) and high tumor budding (grade 3).

### 2.4. Determination of Mismatch Repair Protein Expression

The immunohistochemistry (IHC) method was used to establish the mismatch repair tumor status (MSS or MSI) and to detect the intact or the loss expression of the MMR proteins (MLH1, PMS2, MSH2, and MSH6). The IHC study was performed on unstained formalin-fixed paraffin-embedded (FFPE) tumor tissue sections of 5 *μ*m thickness, on the automated immunostainer Ventana Benchmark ULTRA. We have employed monoclonal antibodies specific for each MMR protein, MLH1 (G168-728/CELL MARQUE), MSH2 (G219-1129/CELL MARQUE), MSH6 (44/CELL MARQUE), and PMS2 (MRQ-28/CELL MARQUE). Adjacent normal tissue (lymphocytes or normal glandular cells) was used as an internal control for positive staining.

### 2.5. Detection of KRAS and NRAS Mutation

#### 2.5.1. DNA Extraction

Genomic DNA was extracted from 5 to 8 sections of 5 *μ*m thickness of macrodissected formalin-fixed paraffin-embedded (FFPE) tumor blocks, containing at least 50% of tumor cells, as determined by an experienced pathologist on H&E-stained paraffin slides. The extraction was effected using the QIAamp DNA FFPE Tissue Kit (Invitrogen) and according to the manufacturer's instructions. DNA concentration (ng/ul) was assessed by Qubit fluorometer.

#### 2.5.2. PCR and Direct Sequencing

For each sample, mutations of *KRAS* exons 2, 3, and 4 and *NRAS* exons 2 and 3 were amplified by polymerase chain reaction (PCR). Briefly, 10 ng of template DNA was amplified using 12× PCR mix platinum, 12.5 pmol primers, 50 *μ*mol Mgcl_2_, and 2.5 *μ*l of the corresponding set of PCR primers listed in Table 1. After the purification of PCR products, the presence of mutations was detected by direct sequencing using the BigDye Terminator V3.1 Cycle Sequencing Kit (ABI Prism) and the Applied Biosystems 3500Dx Genetic Analyzer (Applied Biosystem).

#### 2.5.3. Pyrosequencing

The analysis of RAS mutations was performed using the TheraScreen® KRAS Pyro Kit (for KRAS codons 12 and 13) and the TheraScreen® RAS Extension Pyro Kit (for KRAS codons 59/61, 117, and 146 and NRAS codons 12, 13, 59, 61, 117, and 146) (Qiagen, Germany), according to the manufacturer's instructions. As described previously [2], 5 *µ*l of template DNA (2–10 ng of genomic DNA) was amplified by polymerase chain reaction (PCR) in a 20 *µ*l volume containing 12.5 *µ*l of PyroMark® PCR Master Mix 2x, 2.5 *µ*l of Coral Load Concentrate 10x, 4 *µ*l of nuclease-free water, and 1 *µ*l of the corresponding set of PCR primers (Qiagen). 10 *µ*l of PCR products was immobilized to Streptavidin Sepharose High-Performance beads (Qiagen) to prepare the single-stranded DNA. The corresponding sequencing primers were allowed to anneal to the DNA using a PyroMark Q24 plate and a vacuum workstation (Qiagen). PyroMark Q24 reagents (enzyme mixture, substrate mixture, and nucleotide all from Qiagen) were prepared and loaded into a cartridge to be dispensed during the sequencing process. Finally, the sequences were analyzed using PyroMark Q24 software in the AQ analysis mode. In each run, two controls were included: negative control (without template DNA) and an unmethylated control DNA, provided by the kit as a positive control for PCR, and sequencing reactions were included.

### 2.6. Statistical Analysis

Clinical, pathological, and molecular variables collected at baseline were described as means and standard deviation (sd's) for quantitative variables and percentages for qualitative variables. Associations between tumor budding (assessing as a categorical variable) and categorical factors of tumor were assessed using the *χ*2-test or Fisher's exact test variables. The unpaired *t*-test was used for continuous variables. Tests were statistically significant when *P* < 0.05.

Overall survival was defined as the time from the start of diagnosis until death or until the last follow-up. Relapse-free survival was measured from the date of initial diagnosis until the date of local relapse or regional relapse or last follow-up/death (all causes) whichever occurs first.

RFS and OS rates according to tumor budding, clinicopathological factors, and molecular features were determined using the Kaplan-Meier method, and survival differences between groups were evaluated by log-rank test.

Multivariate analysis was performed using a Cox proportional hazard model to identify independent risk factors for survival. Factors that were significant and nearly significant in univariate analysis (*P* < 0.1) were included in multivariate analysis.

Data from univariate and multivariate analyses were reported as hazard ratios (HRs) with 95% confidence intervals (CIs). All statistics were assessed using 2-sided tests, with *P* values <0.05 considered statistically significant. Statistical analysis was performed using the IBM SPSS Statistics 21.

## 3. Results

### 3.1. Patient Demographics and Pathological Characteristics

Patients and tumor characteristics of 100 patients are summarized in Table 2. Among 100 cases, 43 (43.0%) were women and 57 (57.0%) were men with a mean age of 54.9 years. Our cohort was characterized by a predominance of the left-sided colon cancers (*n* = 62, 62.0%), compared to right-sided CC (*n* = 38, 38.0%). Histologically, the adenocarcinoma subtype was documented in 86 tumors (86.0%), while only 9 (9.0%) tumors were mucinous adenocarcinoma. 46 (46.0%) tumors were classified as grade 2 (moderately differentiated) and grade 1 (well-differentiated), and 8 (8.0%) tumors were classified as grade 3 (poorly differentiated). Perineural invasion was observed in 15 (15.3%) tumors. In this study, the mean number of removed lymph nodes was 20.8 (range, 1–57). 85 (87.6%) patients had more than 12 dissected LN. Positive LNs were identified in 32 (32.6%) patients (mean = 1.3; rang, 0.1–16). According to the TNM classification, 4 (4.2%) of the tumors were stage I, 51 (53.1%) stage II, 25 (26.0%) stage III, and 16 (16.7%) IV. In our cohort, 50% of patients have received surgical treatment, and 46% have received adjuvant chemotherapy. Neoadjuvant treatment was indicated for only 4% of cases.

The mean follow-up time of the patient's OS was 49.4 months (range, 6–119 months). Among 100 patients, 18 (18.2%) cases of death were recorded. Recurrence was observed in 29 (29.3%) patients. The most frequent site of recurrence was local recurrence (31.0%) followed by peritoneums (24.1%), lung (20.7%), and liver (17.4%).

### 3.2. Molecular Features

Concerning molecular characteristics, the MSI tumors were found in 21 patients (21.0%), tumors with KRAS mutations in 31 patients (40.3%), and NRAS mutations in 2 patients (2.6%).

### 3.3. KRAS and NRAS Mutations Classes

All the basic data are presented in Table 3. Activating KRAS mutations were found in 31/77 examined tumor cases (40.3%). Among *KRAS* variants, G12D was the most frequent (11/31, 35.5%), followed by G13D (8/31, 25.8%), G12C (4/31, 12.9%), A146T (3/31, 9.7%), G12V (2/31, 6.5%), G12R (1/31, 3.2%), G12A (1/31, 3.2%), and G13V (1/31, 3.2%).

Out of 77 cases, two showed NRAS mutations (2/77, 2.6%). One mutation was detected in codon 12 (G12R) (1/2, 50%) and the other in codon 61 (Q61L) (1/2, 50%).

### 3.4. Incidence of Tumor Budding in Our Population

Among 100 CC cases, 28 (28%) tumors showed low-grade tumor budding (grade 1), 32 (32%) tumors showed intermediate-grade tumor budding (grade 2), and 40 (40%) tumors showed high-grade tumor budding (grade 3). The results are shown in Table 4.

In correlation analysis, we divided the patients into two groups, a group with low-grade tumor budding (grade 1 + grade 2) that represented 60% of all cases and a group with high-grade tumor budding (grade 3) that represented 40% of all cases (Table 4).

### 3.5. Relationship between Tumor Budding and Clinicopathological Features

Table 5 shows the results of the association between tumor budding and clinicopathologic factors. Tumor budding grades were significantly associated with age, perineural invasion, vascular invasion, distant metastases, TNM stage, the occurrence of relapse, and the number of deceased cases. Indeed, compared with patients with low-grade tumor budding (grade 1 + grade 2), patients with high-grade tumor budding (grade 3) had more vascular invasion (28.2% vs. 15.3%; *P*=0.05), more venous invasion (25.6% vs. 8.5%; *P*=0.02), and a higher number of distant metastases (33.3% vs. 5.0%; *P* < 0.001). Also, these patients had a majority of advanced pathologic stage IV tumors (*P*=0.001).

Interestingly, patients with high-grade tumor budding had significantly high relapse rates (*P*=0.04). Moreover, peritoneal recurrence was the most frequent recurrence site in tumors with high-grade tumor budding, with a difference close to significance (*P*=0.07). Tumors with high-grade tumor budding were correlated with a higher risk of death (*P*=0.02).

There was no correlation between tumor budding grade and gender, tumor localization, histologic subtype, histologic grade, and lymph nodes count.

### 3.6. Tumor Budding and Molecular Biomarkers

Interestingly, we investigated the relationship between the different grades of tumor budding and the molecular characteristics of the tumor. The different results are shown in Table 6. According to our results, tumors with high-grade tumor budding were more likely to be microsatellite stable (MSS) (*P*=0.005).

The mutation rate in the *KRAS* gene was significantly higher in the high-grade TB tumors compared to that in the low-grade TB tumors (*P*=0.02). Tumors with KRAS codon 12 mutations tented to have high tumor budding with a difference close to significance, as compared with tumors harboring other KRAS codon mutations (*P*=0.05). Moreover, *KRAS* G12D mutation was found to be significantly correlated with high-grade TB compared to the other KRAS codon 12 variants (*P*=0.007). However, there was no correlation between KRAS codon 13 variants and tumor budding grade.

There was no significant association between tumor budding and NRAS status.

### 3.7. Survival Outcomes according to Tumor Budding

Table 7 shows associations of tumor budding with overall survival and relapse-free survival of CC patients when tumor budding is stratified into three groups (BD1, BD2, BD3) or two groups (low (BD1 + 2), high (BD3)).

**Figure 2:**
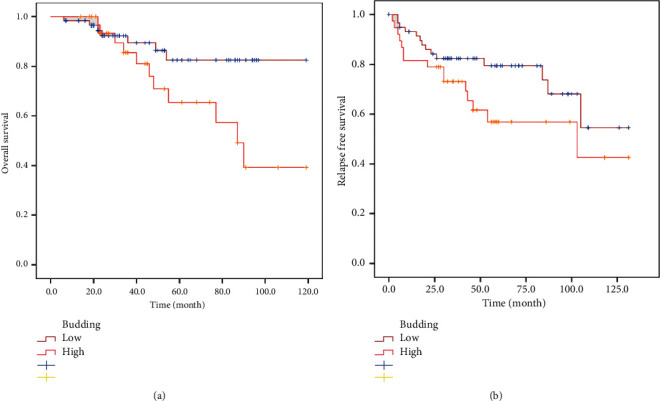
Survival curves of CC patients stratified by budding grades. (a) Overall survival in all patients stratified by BD1 + 2 (low) versus BD3 (high). (b) Relapse-free survival in all patients stratified by BD1 + 2 (low) versus BD3 (high).

In the three-tier analysis, tumor budding was not associated with OS and RFS (BD1 versus BD2 versus BD3). In the 2-tier approach (BD1 + 2 versus BD3), tumors with high-grade tumor budding were significantly correlated with shorter OS (*P*=0.04; Figure 2(a)). Moreover, these tumors tended to be associated with shorter RFS with a difference close to significance (*P*=0.09; Figure 2(b)).

## 4. Discussion

The present study was designed to investigate the relationship of tumor budding with the clinicopathological characteristics and molecular biomarkers of CC. Also, we evaluate the prognostic impact of tumor budding on hundred CC patients using the ITBCC scoring method of TB on HES slides for the first time in the Moroccan population and the Middle East and Nord Africa region.

In recent years, several reports showed that tumor budding is characterized by different clinicopathological and histological features. In this study, we were able to demonstrate that high-grade tumor budding (BD3) grade underlines special clinicopathological parameters. In our context, BD3 was significantly greater in older age patients and we are the first to report this result because none of the previous studies have found a significant association between age and tumor budding grades [5, 12, 13].

As reported in many studies [5, 10, 14], we documented a positive relationship between high-grade tumor budding, the presence of vascular invasion, and the presence of perineural invasion. Furthermore, we found that tumors with high-grade tumor budding were significantly characterized by an increased frequency of distant metastases. The same result was reported by Jayasinghe et al. [15]. These associations have fueled the hypothesis that tumor buds can pervade the extracellular matrix (ECM) and migrate and disseminate into blood vessels [16]. It was suggested that tumor budding harbors the properties of cells undergoing an epithelial-mesenchymal transition (EMT) or a partial-EMT state [16]. In this process, epithelial cells lose intracellular and cell-matrix contacts mediated by E-cadherin, leading to invasion and metastatic cancer spread [17].

Additionally, we observed a significant correlation between high-grade tumor budding and advanced TNM stage, which further supports the results of previous studies as reported by Van et al. [18].

Interestingly, our results indicate that patients exhibiting high-grade tumor budding had a higher rate of recurrence. In a study conducted on 138 patients, Tanaka et al. [19] reported that tumor budding was significantly associated with disease recurrence. Another study evaluated 200 patients with CC and reported the same result [20]. Okuyama et al. showed a statistically significant relationship between high-grade tumor budding and local recurrence [21].

The majority of studies investigating the relationship between tumor budding and clinicopathological features have documented its positive correlation with lymph node involvement [22]. Conversely, in our study, we did not find any association.

As a point of interest, we also investigated the association between tumor budding and molecular biomarkers, such as MSI status, KRAS, and NRAS mutations. We observed that high-grade tumor budding was more common in MSS tumors, which is consistent with findings reported by previous studies [12, 23, 24]. In addition, Lugli et al. have recently showed that tumor buds are infrequently found in colorectal cancers with microsatellite instability (MSI) [7]. It was showed that MSS tumors were significantly correlated with shorter overall survival of CC patients [25].

We found that tumors with high-grade tumor budding had significantly more KRAS mutations. The relationship between KRAS mutation and tumor budding has previously been reported [26, 27]. Jang and colleagues found that 61.8% of colorectal cancers with high-grade tumor budding harbor more KRAS mutations [28]. Recently, Trinh et al. [9] and Lugli et al. have reported the same results [7]. Similar to Jang et al. [28], we found that the G12D substitution in the KRAS gene was strongly associated with high-grade tumor budding. According to this result, the KRAS G12D mutation could be proposed as a high-grade tumor budding biomarker.

Of note, our group has previously reported KRAS mutations as a predictor factor of worse OS [2].

We did not find any association between NRAS status and tumor budding grade in our context. Considering we found only 2 patients harboring NRAS mutations, this finding does not yet allow us to draw firm conclusions. Barresi et al. were also limited by a small number of NRAS mutated cases (*N* = 4) and they did not find any correlation between NRAS status and tumor budding grade [27].

According to our results, it can be reported that high grade of tumor budding is generally associated with poor prognostic factors (vascular and perineural invasion, distant metastases, MSS status, and KRAS mutations).

Secondly, we aimed to evaluate the relationship between tumor budding and clinical outcome in CC patients, for the first time in the Middle East and North Africa region. We validated the prognostic effect of tumor budding on overall survival in our cohort using the newly established ITBCC criteria for the scoring of tumor budding on H&E slides. We found that OS was better in patients with low-grade tumor budding (BD1/2 versus BD3) at all stages; we also observed that high-grade tumor budding was linked to an increased risk of death. This method is already included in the Japanese Guidelines for the reporting of CRC. However, many reports suggest that whichever scoring method is utilized, the presence of high-grade tumor budding is correlated with worse clinical outcomes [10, 29, 30].

In the literature, several studies are investigating the impact of tumor budding on CC patient's survival. Similar to our results, Oh and colleagues pooled results from more than 4000 Japanese patients from all stages and confirmed the positive association of high-grade budding with worse OS [31]. Trinh et al. also validated the prognostic impact of tumor budding independent of age, stage, and sex in a cohort including 1320 colorectal cancers [9].

Regarding the association between tumor budding and RFS, we observed that patients with high tumor budding had a shorter RFS but with a difference close to significant although several reports have confirmed this correlation with significant differences [31, 32].

A preprint has previously been published [33].

There were some limitations to the present study. First is the size of the study cohort. Indeed, the number of cases included in our study is relatively small, in comparison with some previous reports. Second, our study represents a single institution and thus carries the possibility of selection bias and does not allow us to generalize our results in the overall population of our country. Third, it has been demonstrated that high-grade tumor budding is significantly associated with lymph node metastasis in colon cancer. However, we were unable to produce similar results. This could be likely attributable to the size and the characteristics of our sample.

Above all, our study is the first report investigating the prognostic impact on colon cancer patients in the Middle East and Nord Africa region.

## 5. Conclusions

In this study, we concluded that high-grade tumor budding was strongly associated with unfavorable clinicopathological features, like a perineural invasion, venous invasion, and distant metastases, and special molecular biomarkers which are MSS status and KRAS mutations. We defined KRAS G12D mutation as a biomarker of high-grade tumor budding.

Our results also indicate that high-grade tumor budding effectively affects the overall survival of CC patients. Based on these findings and the ITBCC group recommendations, tumor budding should be taken into account along with other clinicopathologic factors in the risk assessment of colorectal cancer.

## Figures and Tables

**Table 1:** Primer sequences used for PCR.

Primer name	Primer sequence
KRAS-ex 2- F	5′-GGTGGAGTATTTGATAGTGTA- 3′
KRAS-ex 2- R	5′-TGCATATTACTGGTGCAGACC- 3′
KRAS-ex 3- F	5′-AGTAAAAGGTGCACTGTAATAA-3′
KRAS-ex 3- R	5′-ATAATAAGCTGACATTAAGGAG-3′
KRAS-ex 4- F	5′-TGTTACTAATGACTGTGCTATAACTTTT-3′
KRAS-ex 4- R	5′-TATGCTATACTATACTAGGAAATAAAA-3′
NRAS-ex2-F	5′-ATGACTGAGTACAAACTGGTGGTGGTTGGAGCA-3′
NRAS-ex2-R	5′-CACTTTGTAGATGAATATGATCCCACCATAGAG-3′
NRAS-ex3-F	5′-GATTCTTACAGAAAACAAGTGGTTA-3′
NRAS-ex3-R	5′-CATTTGCGGATATTAACCTCTACAG-3′
NRAS-ex4-F	5′-GGAGCAGATTAAGCGAG-3′
NRAS-ex4-R	5′-TCAGCCAAGACCAGACAG-3′

**Table 2:** Patient demographics, pathological, and molecular features.

Characteristics	Total (%)
Age	
≤57	53 (53.0%)
≥57	47 (47.0%)
Gender	
Female	43 (43.0%)
Male	57 (57.0%)
Tumor site	
Right colon	38 (38.0%)
Left colon	62 (62.0%)
Histological subtype	
Adenocarcinoma	86 (86.0%)
Mucinous adenocarcinoma	9 (9.0%)
Others	5 (5.0%)
Histological grade	
Well	46 (46.0%)
Moderate	46 (46.0%)
Poor	8 (8.0%)
Venous invasion	
Presence	20 (20.4%)
Absence	78 (79.6%)
Perineural invasion	
Presence	15 (15.3%)
Absence	83 (84.7%)
Number of removed lymph nodes	
Mean (±SD)	20.8 (±10.8)
<12	12 (12.4%)
≥12	85 (87.6%)
Range	1–57
Positive lymph node	
Mean (±SD)	1.3 (±2.9)
Presence	32 (8.6%)
Absence	66 (32.7%)
Average	0.1–16
Lymph node ratio	
Mean (±SD)	0.1 (±0.14)
Range	0.01–0.81
Distant metastases (M)	
M0	83 (83.8%)
M1	16 (16.2%)
Disease stages	
I	4 (4.2%)
II	51 (53.1%)
III	25 (26.0%)
IV	16 (16.7%)
Treatment	
Surgery	50 (50.0%)
Surgery + adjuvant chemotherapy	46 (46.0%)
Neoadjuvant therapy	4 (4.0%)
Follow-up time (months)	
Mean (SD)	49.4 (±29.3)
Range	6–119
Recurrence	
(+)	29 (29.3%)
(−)	70 (70.7%)
Recurrence patterns	
Liver	5 (17.4%)
Lung	6 (20.7%)
Peritoneum	7 (24.1%)
Local recurrence	9 (31.0%)
Others	2 (6.8%)
Mortality	
Death cases	18 (18.2%)
Censored cases	81 (81.8%)
MSI status	
MSS	79 (79.0%)
MSI	21 (21.0%)
KRAS mutation	
Presence	31 (40.3%)
Absence	46 (59.7%)
NRAS mutation	
Presence	2 (2.6%)
Absence	75 (97.4%)

**Table 3:** The frequencies of genetic alteration classes.

Mutations	Number	%
KRAS	31	40.3
Codon 12	19	61.3
G12D	11	35.5
G12C	4	12.9
G12V	2	6.5
G12A	1	3.2
G12R	1	3.2
Codon 13	9	29.0
G13D	8	25.8
G13V	1	3.2
Codon 146	3	9.7
A146T	3	9.7

NRAS	2	2.6
Codon 12	1	50
G12R	1	50
Codon 61	1	50
Q61L	1	50

**Table 4:** Incidence of tumor budding.

Tumor budding grades	Frequency (%)
Grade 1 (low)	28 (28%)
Grade 2 (intermediate)	32 (32%)
Grade 3 (high)	40 (40%)
Low-grade tumor budding	60 (60%)
High-grade tumor budding	40 (40%)

**Table 5:** Association between tumor budding and clinicopathological features.

Variables	Low-grade tumor budding	High-grade tumor budding	*P* value
Age			0.03
<57	36 (60.0%)	17 (42.5%)	
≥57	24 (40.0%)	23 (57.5%)	
Genre			0.2
Female	28 (46.7%)	15 (37.5%)	
Male	32 (53.3%)	25 (62.5%)	
Tumor site			0.4
Right colon	22 (36.7%)	16 (40.0%)	
Left colon	38 (63.3%)	24 (60.0%)	
Histologic subtype			0.2
Adenocarcinoma	54 (90.0%)	32 (80.0%)	
Mucinous	3 (5.0%)	6 (15.0%)	
Others	3 (5.0%)	2 (5.0%)	
Histologic grade			0.5
Well	28 (46.7%)	18 (45.0%)	
Moderate	26 (43.3%)	20 (50.0%)	
Poor	6 (10.0%)	2 (5.0%)	
Venous invasion			0.05
Presence	9 (15.3%)	11 (28.2%)	
Absence	50 (84.7%)	28 (71.8%)	
Perineural invasion			0.02
Presence	5 (8.5%)	10 (25.6%)	
Absence	54 (91.5%)	29 (74.4%)	
Number of removed lymph nodes			
Mean (SD)	21.7 (±8.5)	19.2 (±6.9)	0.2
˂12	5 (8.6%)	7 (17.9%)	0.1
˃12	53 (91.4%)	32 (82.1%)	
Positive lymph node			
Mean (SD)	1.2 (±0.0)	1.4 (±2.9)	0.8
Presence	19 (32.2%)	13 (33.3%)	0.5
Absence	40 (67.8%)	26 (66.7%)	
RGL			
Mean (SD)	0.06 (±0.03)	0.063 (±0.2)	0.9
Synchronous metastasis (M)			˂0.001
M0	57 (95.0%)	26 (66.7%)	
M1	3 (5.0%)	13 (33.3%)	
Disease stages			0.001
I	2 (3.3%)	2 ((5.0%)	
II	37 (61.7%)	15 (37.5%)	
III	18 (30.0%)	9 (22.5%)	
IV	3 (5.0%)	14 (35.0%)	
Follow-up time (months)	27.0 (±19.3)	24.8 (±13.3)	0.7
Recurrence			
(+)	14 (23.3%)	15 (38.5%)	0.04
(−)	46 (76.7%)	24 (61.5%)	
Recurrence patterns			
Liver	2 (14.3%)	3 (20%)	0.07
Lung	3 (21.4%)	3 (20%)	
Peritoneum	2 (14.3%)	5 (33.3%)	
Local recurrence	6 (42.9%)	3 (20%)	
Others	1 (7.1%)	1 (6.7%)	
Mortality			0.02
Death cases	7 (11.7%)	11 (28.2%)	
Censored cases	53 (88.3%)	28 (71.8%)	

**Table 6:** Association between tumor budding and molecular biomarkers.

Variables	Low-grade tumor budding	High-grade tumor budding	*P* value
MSI status			0.005
MSS	42 (70.0%)	37 (92.5%)	
MSI	18 (30.0%)	3 (7.5%)	
KRAS status			
Mutant	14 (23.3%)	17 (42.5%)	0.02
Wild-type	46 (76.7%)	23 (57.5%)	
KRAS codon types			
Codon 12	7 (50%)	12 (70.6%)	0.05
Codon 13	5 (35.7%)	4 (23.5%)	
Codon 146	2 (14.3%)	1 (5.9%)	
KRAS codon 12 variants			
G12D	1 (14.3%)	10 (83.3%)	0.007
G12C	2 (28.6%)	2 (16.7%)	
G12V	2 (28.6%)	0 (0.0%)	
G12A	1 (14.3%)	0 (0.0%)	
G12R	1 (14.3%)	0 (0.0%)	
KRAS codon 13 variants			
G13D	4 (80%)	4 (100%)	0.3
G13V	1 (20%)	0 (0.0%)	
NRAS gene status			0.4
Mutant	1 (1.7%)	1 (2.5%)	
Wild-type	59 (98.3%)	59 (97.5%)	

**Table 7:** Analysis of OS and RFS according to tumor budding.

Tumor budding	Mean OS month (95% CI)	*P* value	Mean RFS month (95% CI)	*P* value
BD1	85.7 (76.9–94.5)	0.1	88.7 (69.7–107.7)	0.1
BD2	100.5 (84.0–116.9)		108.4 (90.8–126.1)	
BD3	82.6 (67.2–97.9)		81.4 (62.7–100.1)	

High	82.5 (67.2–97.8)	0.04	81.4 (62.7–100.1)	0.09
Low	104.2 (94.0–114.4)		99.4 (80.9–103.6)	

## Data Availability

The datasets used/or analyzed during the current study are available from the corresponding author on reasonable request.
